# Rare-Earth-Modified Titania Nanoparticles: Molecular Insight into Synthesis and Photochemical Properties

**DOI:** 10.1021/acs.inorgchem.1c02134

**Published:** 2021-09-13

**Authors:** Fredric G. Svensson, Bogdan Cojocaru, Zhen Qiu, Vasile Parvulescu, Tomas Edvinsson, Gulaim A. Seisenbaeva, Carmen Tiseanu, Vadim G. Kessler

**Affiliations:** † Department of Molecular Sciences, Swedish University of Agricultural Sciences, Box 7015, Uppsala SE-75007, Sweden; ‡ Department of Chemistry, University of Bucharest, B-dul Regina Elisabeta, No. 4−12, Bucharest RO-030018, Romania; § Department of Materials Science and Engineering, Uppsala University, Box 53, Uppsala SE-75103, Sweden; ∥ National Institute for Laser, Plasma and Radiation Physics (NILPR), Bucharest-Magurele RO-76900, Romania

## Abstract

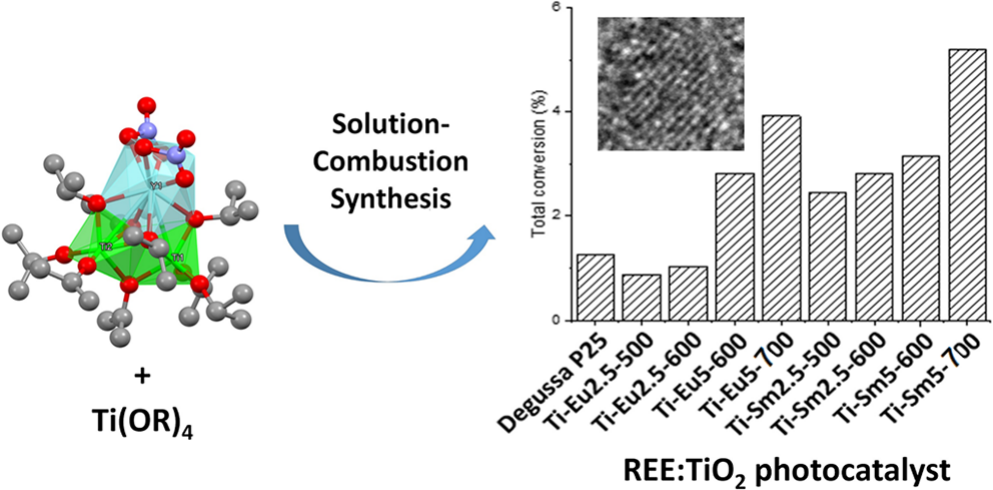

A molecular precursor approach to titania (anatase) nanopowders modified with different amounts of rare-earth elements (REEs: Eu, Sm, and Y) was developed using the interaction of REE nitrates with titanium alkoxides by a two-step solvothermal–combustion method. The nature of an emerging intermetallic intermediate was revealed unexpectedly for the applied conditions via a single-crystal study of the isolated bimetallic isopropoxide nitrate complex [Ti_2_Y(^*i*^PrO)_9_(NO_3_)_2_], a nonoxo-substituted compound. Powders of the final reaction products were characterized by powder X-ray diffraction, scanning electron microscopy–energy-dispersive spectroscopy, Fourier transform infrared, X-ray photoelectron spectroscopy, Raman spectroscopy, and photoluminescence (PL). The addition of REEs stabilized the anatase phase up to ca. 700 °C before phase transformation into rutile became evident. The photocatalytic activity of titania modified with Eu^3+^ and Sm^3+^ was compared with that of Degussa P25 titania as the control. PL studies indicated the incorporation of Eu and Sm cations into titania (anatase) at lower annealing temperatures (500 °C), but an exclusion to the surface occurred when the annealing temperature was increased to 700 °C. The efficiency of the modified titania was inferior to the control titania while illuminated within narrow wavelength intervals (445–465 and 510–530 nm), but when subjected to a wide range of visible radiation, the Eu^3+^- and Sm^3+^-modified titania outperformed the control, which was attributed both to doping of the band structure of TiO_2_ with additional energy levels and to the surface chemistry of the REE-modified titania.

## Abstract 2

Molecular insight into the mechanism of solution−combustion synthesis provided clues to understanding the photocatalytic properties of rare-earth-doped nanotitania.

## Introduction

Titania (TiO_2_) is one of the most investigated semiconductor nanomaterials, primarily because of its photocatalytic activity, chemical stability, low toxicity, and facile synthesis.^1,2^ The discovery of its ability to split water in the 1970s by Fujishima and Honda^3^ encouraged intensive research for applications in the photocatalytic degradation of organic pollutants^4^ and hydrogen production from water.^5^ The two most common phases of titania are anatase and rutile. Anatase is considered to be the catalytically most active phase.^6^ Still, the band gap of anatase is ca. 3.2 eV, and UV radiation is therefore required to promote an electron from the valence band (VB) to the conduction band (CB). This makes photocatalysis in sunlight inefficient. To overcome this obstacle, different strategies, including sensitizing and doping, have been employed to make use of visible light for photocatalysis. In sensitizing, an organic or inorganic compound is attached to the surface of titania. This compound absorbs in the visible spectrum and can transfer an excited electron into the CB of titania.^7−9^ This is used in dye-sensitized solar cells.^10^ Doping of metals or nonmetals into the titania lattice may generate intermediate energy levels between the VB_TiO_2__ and CB_TiO_2__ and redshift the absorption spectrum into the visible region. For substitutional doping of titania, it is a requirement that the cation that replaces Ti has a similar ionic radius.

There has been recent interest in titania “doped” with rare-earth elements (REEs).^11−14^ The f orbitals of the REE “dopants” can act as electron traps, thereby decreasing the electron–hole recombination rates, and facilitate transfer to surface hydroxide groups to form reactive hydroxyl radicals. An increased efficiency from Gd^3+^ doping was explained by its stable 4f^7^ configuration with half-filled f orbitals. Transfer of an electron from VB_TiO_2__ to one of the half-filled Gd f orbitals would cause decreased stability. To regain the more stable configuration of half-filled f orbitals, the excited electron will be kicked out and has the possibility of reaching the particle surface.^12,15^ A suggested mechanism of this is that charge imbalance upon doping and the locally created permanent dipoles would facilitate more efficient charge separation upon excitation and subsequent charge transport.^16^ An additional benefit of REE “doping” of titania is the formation of surface complexes between organic molecules with free electron pairs (i.e., those containing O, N, or S) and the REEs.^17^ The hydroxyl radicals on the particle surface are responsible for the degradation of organic molecules, and an improved surface adsorption capacity would result in more efficient degradation.^11,12,18^ There appears to be an optimum amount of REE “dopants” because at a certain concentration the catalytic activity starts to decrease. This has been suggested to depend on the formation of recombination centers and active site blocking.^15,19,20^ The atomic radii^21^ of the lanthanide (Ln) ions (about 1.10–1.20 Å) are considerably larger than that of Ti^4+^ (0.745 Å), and “doping” may instead result in surface-bound REEs, REE oxides (RE_2_O_3_), mixed phases (e.g., REE titanates), or intercalation. Different mixed-metal titanates have previously been demonstrated to function as inorganic sensitizers by interfacial charge transfer to titania,^22,23^ and it is likely that REE titanates and REE oxides can do the same, although this appears to be less investigated. It is generally observed that the addition of REE “dopants” results in smaller average particle size and delayed anatase-to-rutile phase transformation.^20,24^ Using conventional low-temperature sol–gel processing for doping titania with Ln ions (and other elements with substantially larger ionic radii) may lead to the segregation of a lanthanide oxide phase on the titania surface,^25,11^ and the hydrothermal synthesis of REE-doped titania can result in mixed anatase–rutile or anatase–brookite phases.^26,27^

In this work, we focused on the development of a molecular precursor approach to titania nanoparticles modified with three different REEs (Y, Sm, and Eu) using a solvothermal method with subsequent annealing at different temperatures. Our aim was to bring a molecular understanding to how REE can be incorporated into the titania structure both in the course of the reaction and with respect to their occurrence in the final oxide products. Although yttrium is classified as a REE, it has a somewhat smaller radius (1.04 Å for Y^3+^). It was applied in isolation of the molecular model for the intermediate species in the synthesis. Eu and Sm are positioned next to each other in the Periodic Table and have similar ionic radii (1.087 and 1.098 Å, respectively, in their 3+ oxidation states). Photoluminescence (PL) of Eu and Sm was employed to determine the positions of the Ln ions in the resulting titania materials.

## Materials and Methods

### Chemicals

Titanium(IV) ethoxide (Sigma-Aldrich), titanium(IV) isopropoxide (Sigma-Aldrich), Y(NO_3_)_3_·6H_2_O (99.8%, Aldrich), Sm(NO_3_)_3_·6H_2_O (99.9%, Aldrich), and Eu(NO_3_)_3_·5H_2_O (99.9%, Aldrich) were used as received. Ethanol (99.7%, Solveco) was refluxed and distilled over metallic calcium, and isopropyl alcohol (Sigma-Aldrich) was refluxed and distilled over lithium aluminum hydride.

### Preparation of REE-Modified Nanopowders and the Molecular Intermediate

The appropriate amounts of REE nitrates (i.e., 2.5 or 5 mol % with respect to Ti) were dissolved in anhydrous ethanol in Teflon containers. Titanium(IV) ethoxide was then added under a nitrogen atmosphere in a glovebox. The Teflon containers were placed in steel autoclaves and treated as follows: ramped at 2.6 °C min^–1^ to 60 °C (held for 4 h) and then ramped at 3.3 °C min^–1^ to 160 °C (held for 20 h). Brownish transparent liquids were obtained. After drying at 45 °C, brown-orange, amorphous powders were produced. The powders were ground in an agate mortar and annealed at different temperatures (400–800 °C in increments of 100 °C). Ramping from room temperature to the final temperature was set to 1 h, and this temperature was held for 2 h. ***Caution!**The presence of nitrates can cause ignition upon heating with small pieces that can be spread around! Heating should be carried out in a closed porcelain cup and the door of the oven kept properly closed until the end of the process!* The powders were cooled naturally to room temperature in the oven. The different powders were designated as Ti-REE*x*-*t*, where REE is Y, Sm, or Eu, *x* is either 2.5 or 5 mol %, and *t* is the annealing temperature.

The molecular intermediate, Ti_2_Y(^*i*^PrO)_9_(NO_3_)_2_ (**1**), was synthesized by a solvothermal method. To 0.80 mL of anhydrous toluene containing 0.15 equiv of Y(NO_3_)_3_·6H_2_O was added 0.51 mmol of Ti(^*i*^PrO)_4_ under a nitrogen atmosphere. The reaction mixture was placed in a Teflon-lined steel autoclave, heated to 85 °C for 44 h, and then naturally cooled to room temperature to obtain small colorless needle-shaped crystals of compound **1**. Crystallographic details are presented in Table 1. The data were collected at room temperature using Mo Kα radiation, λ = 0.71073 Å, with a Bruker D8 SMART Apex2 diffractometer. The structure was solved by direct methods. The coordinates of the metal atoms were obtained from the initial solution, and those of other atoms were found in subsequent Fourier syntheses. All non-H atoms were refined first in isotropic and then in anisotropic full-matrix approximations. H atoms were added via geometric calculation and included in the final refinement in the isotropic approximation. Nitrate ligands were severely disordered, and possible alternative configurations of O atoms were introduced using partial occupation. Full details of the structure solution and refinement are available from the Cambridge Crystallographic Data Center at https://ccdc.cam.ac.uk/ using CCDC 2095265.

**Table 1:** Crystallographic Data for Compound **1**

chemical composition	C_27_H_63_N_2_O_15_Ti_2_Y
fw (g mol^–1^)	840.50
cryst syst	triclinic
space group	*P*1̅
R1	0.0716
wR2	0.1711
GOF	1.112
*a* (Å)	11.653(6)
*b* (Å)	11.700(6)
*c* (Å)	16.983(8)
α (deg)	95.621(6)
β (deg)	101.909(6)
γ (deg)	116.690(5)
*V* (Å^3^)	1975.6(16)
*T* (K)	296(2)
*Z*	2
no. of reflns	4672
data completeness	0.991
CCDC	2095265

Compound **1** was not stable over time, even in a freezer, but decomposed within a few days, possibly as a result of the redox sensitivity of the ^*i*^PrO ligands.

### Characterization

Powders were characterized by scanning electron microscopy (SEM; Hitachi TM-1000 and Hitachi FlexSEM 1000II), energy-dispersive X-ray spectrometry (EDS; Oxford Instruments), powder X-ray diffraction (PXRD; Bruker D8 Apex II CCD diffractometer, Mo Kα, λ = 0.71073 Å, with a graphite monochromator). The PXRD data were treated and analyzed with the Bruker *APEX2* program suite and *EVA v12*) and Fourier transform infrared (FTIR; PerkinElmer Spectrum 100). For atomic force microscopy (AFM), a Bruker Dimension Fastscan atomic force microscope with ScanAsyst was used. Raman spectroscopy, PL, and photocatalysis were performed at the NILPR (Romania). The luminescence measurements were carried out using a Fluorolog 3 spectrofluorometer (Horiba) operated in a fluorescence mode. The luminescence spectra were measured upon excitation into the broad UV absorption of TiO_2_ (Sm) or Ln f–f absorption transitions (Eu). A JEOL JEM-2100F transmission electron microscope was used for transmission electron microscopy (TEM) imaging at a 200 kV operating voltage. X-ray photoelectron spectroscopy (XPS) was measured on a PHI Quantum 2000 spectrometer with monochromated Al Kα radiation with a 45° angle of electron emission. The survey scan spectra were recorded from 0 to 1100 eV (binding energy range), using 224 eV pass energy and 0.8 eV step^–1^. The high-resolution XPS spectra for Ti 2p, O 1s, and C 1s were performed with a pass energy of 55 eV and a step energy of 0.05 eV. Electron and ion neutralization was used for all measurements. The resulting spectra were analyzed by *CasaXPS* software. All of the peaks were calibrated with adventitious C 1s with a binding energy of 284.8 eV.

## Results and Discussion

Using a molecular precursor with known stoichiometry of metal ions, i.e., a single-source precursor, is an attractive way to ensure homogeneous distribution of the dopant in the oxide during hydrolysis/thermolysis, compared to annealing of the separate oxides, carbonates, etc. Solution–combustion synthesis is well reported, for instance, in the synthesis of different bimetallic oxides using the polymeric precursor synthesis. A common example is the complexation of metal ions with citric acid.^28−30^ Mixing the different metal ion–citric acid complexes then provides a very homogeneous mixture of the metal ions in the reaction mixture. Commonly, an oxidizer such as nitrate is also introduced. During the annealing step, the organic part is combusted with the emergence of a homogeneous mixed-metal oxide phase, often of high phase purity. In this work, we wanted to explore a less common method to REE-modified titania: combustion of solvothermally synthesized intermediates from the reaction between titanium alkoxides and REE nitrates, utilizing the alkoxide ligands as fuel and nitrate as an oxidizer. However, only for yttrium could a bimetallic precursor (compound **1**) be isolated when changing titanium(IV) ethoxide for titanium(IV) isopropoxide, allowing one to shed light on the nature of chemical intermediates.

### Synthesis of Bimetallic Complex

Attempts were made to synthesize REE-Ti mixed-metal (oxo)alkoxide complexes. These can be employed as single-source precursors to obtain doped/bimetallic oxides. The advantage here is an even distribution of elements already present in the precursors, which can facilitate an even distribution of the dopant in the final oxide, which is expected to lead to improved photochemical properties. The synthesis of complexes between titanium(IV) alkoxides and nitrates of Y, Eu, and Sm was attempted without introducing any additional organic ligands.

**Figure 1:**
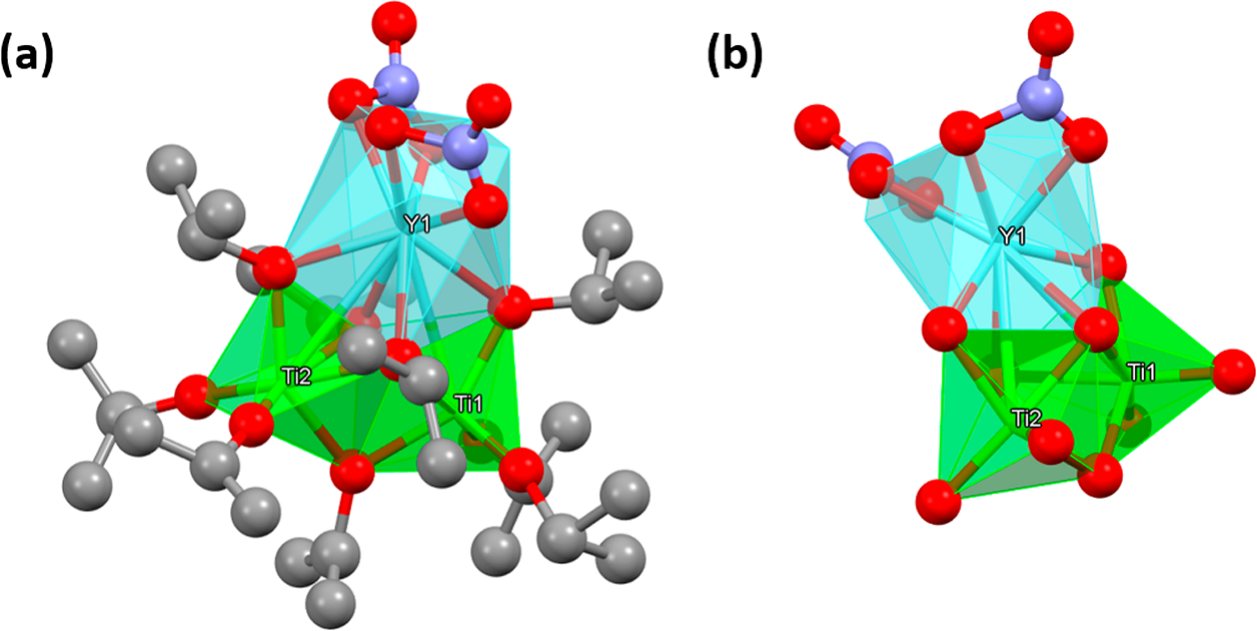
(a) Molecular structure of compound **1**. H atoms and disordered O atoms in the nitrate groups have been omitted for clarity. (b) Metal–oxo core of compound **1**. Color code: green, Ti; turquoise, Y; purple, N; red, O; gray, C.

From the solvothermal method, one mixed titanium–yttrium alkoxide, compound **1**, was obtained (Figure 1). Compound **1** crystallized in the triclinic space group *P*1̅ as transparent needle crystals. Crystallographic data are given in Table 1. The crystals were moisture-sensitive and quickly hydrolyzed without protection. Compound **1** has a triangular structure containing a commonly observed Ti_3_O fragment^31^ but with one Ti exchanged for Y. Notably, the complex contains no oxo bridges despite the presence of water from Y(NO_3_)_3_·6H_2_O. This is presumably due to the lower solubility of complex **1** in the hydrophobic toluene solvent. When adding more than 0.30 equiv of Y(NO_3_)_3_·6H_2_O to Ti, only white precipitates were obtained. Ti and Y are linked via bridging isopropoxide groups. The average bond length for terminal isopropoxide groups is 1.754 Å (sd = 0.0051 Å), while the average bond length for the isopropoxide groups bridging the two Ti centers is 2.109 Å (sd = 0.097 Å). The Ti–^*i*^PrO and ^*i*^PrO–Y bond lengths are asymmetric, with average bond lengths of 2.324 and 1.9735 Å for Y–^*i*^PrO and Ti–^*i*^PrO, respectively. A number of bimetallic oxoalkoxide complexes between Ti and REEs have been reported.^32−34^ These, however, are commonly partially hydrolyzed, contain oxo bridges, and are modified with ligands such as carboxylates. A structural analogue, [Ti_2_Y(^*i*^PrO)_9_Cl_2_], to compound **1** was previously reported by Veith and co-workers^35^ but produced starting with anhydrous yttrium chloride.

**Figure 2:**
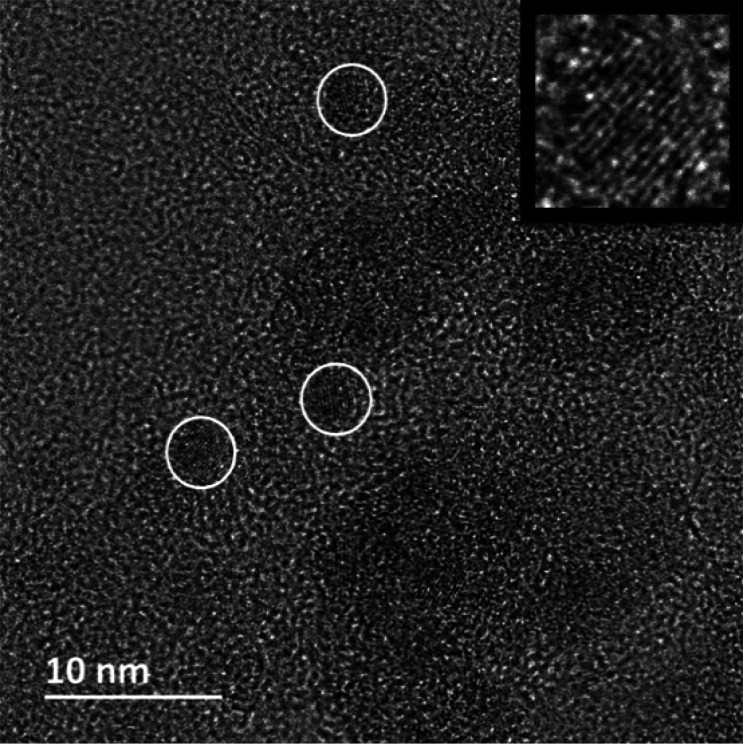
HRTEM micrograph of **1** treated with water. A few primary particles with crystalline cores (anatase) are indicated. The diameter of the particles is ca. 3 nm. Inset: Increased magnification of an anatase nucleus.

We have previously investigated the hydrolysis of a range of titanium alkoxide complexes containing different organic ligands, including the carboxylate, phenoxide, and phosphonate groups.^36−38^ In all cases, the complexes hydrolyzed into titania (anatase). It was interesting to investigate the hydrolysis product of compound **1** because bimetallic titanium alkoxide complexes can serve as precursors of both doped titania^39^ and bimetallic oxides.^40^ Crystals were treated with deionized water and analyzed by high-resolution transmission electron microscopy (HRTEM). Compound **1** formed uniform spherical crystallites belonging to the nondoped anatase phase of titania, according to the lattice fringe distance of 0.2 nm, which corresponds to the 200 plane of anatase (Figure 2) and EDS analysis. This approach was thus not suitable for the production of target materials. On the contrary, annealing of dried powders produced by solvothermal synthesis with subsequent drying and regrinding offered doped samples of anatase after subsequent annealing, as described in the experimental section.

### PXRD

**Figure 3:**
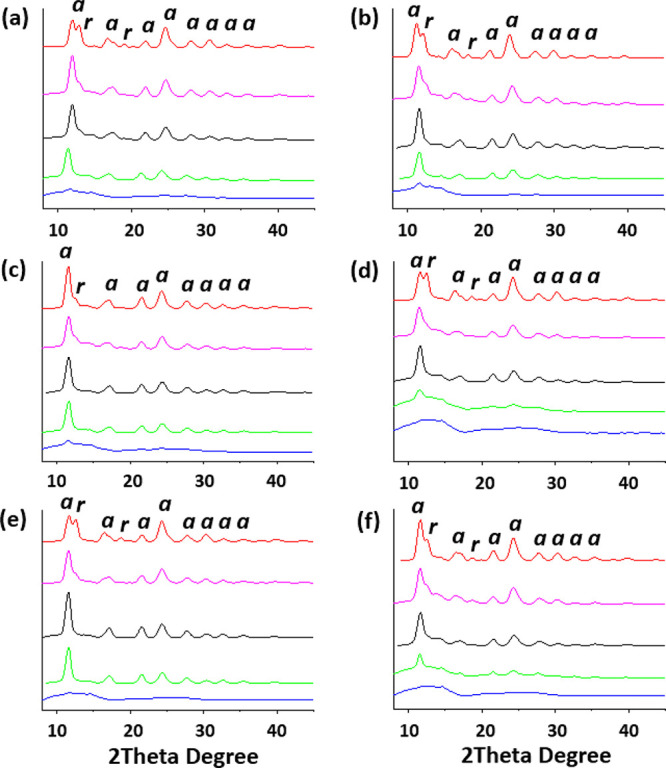
PXRD patterns of the REE-modified titania nanopowders at different annealing temperatures: (a) Ti-Y2.5; (b) Ti-Y5; (c) Ti-Eu2.5; (d) Ti-Eu5; (e) Ti-Sm2.5; (f) Ti-Sm5. Red, magenta, black, green, and blue represent annealing temperatures of (top to bottom) 800, 700, 600, 500, and 400 °C, respectively. Peaks labeled “*a*” and “*r*” belong to the anatase and rutile phases, respectively.

The annealed titania nanopowders were characterized by PXRD, and the diffractograms for 400, 500, 600, 700, and 800 °C are presented in Figure 3. Annealing at 400 °C was not enough to transform the amorphous titania to anatase. All samples modified with 2.5 mol % REE had transformed into titania during annealing at 500 °C. For the 5 mol % samples, only Ti-Y5-500 had transformed into anatase, while Ti-Eu5-500 and Ti-Sm5-500 were mostly amorphous but with traces of anatase. This is consistent with previous reports that an increased amount of dopants retards crystallization. The presence of Ti-O-REE bonds in the amorphous particles would complicate crystallization into anatase and phase transformation into rutile.^24,41^ The reason Ti-Y5-500 could transform into anatase at 500 °C but not Ti-Eu5-500 and Ti-Sm5-500 may be a consequence of the size differences. Because Y^3+^ has a smaller ionic radius, reorganization into anatase might be easier compared to that for the larger Sm^3+^ and Eu^3+^. Both 2.5 and 5 mol % modified powders annealed at 600 °C displayed phase-pure anatase, while the annealed powders at 700 °C mostly consisted of anatase but with traces of rutile. At 800 °C, anatase is still the major phase, but it is evident that transformation into rutile has begun. The anatase-to-rutile transformation is generally initiated from the particle surface,^42^ and the presence of Ti-O-REE bonds on the surface would aggravate this process. It is possible that the deposition of REE oxides or REE titanates onto the titania particle surfaces was able to help in stabilization of the anatase.

### SEM and AFM

**Figure 4:**
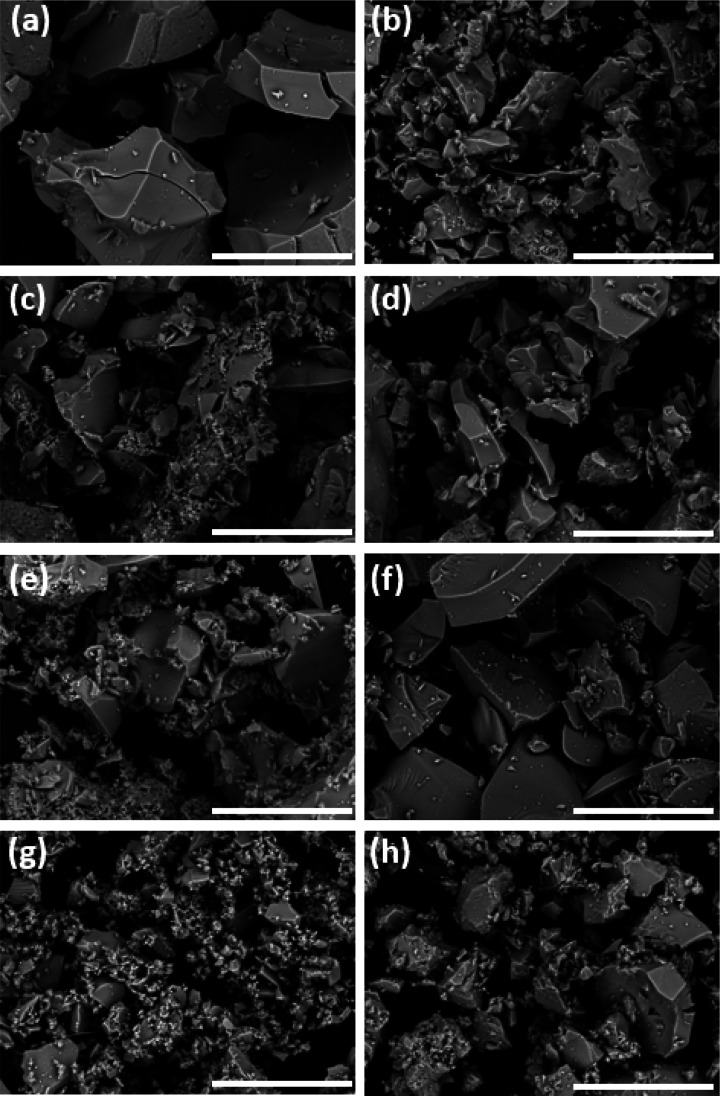
SEM micrographs of the TiO_2_-REE powders used for catalysis: (a) Ti-Eu2.5-500; (b) Ti-Sm2.5-500; (c) Ti-Eu2.5-600; (d) Ti-Sm2.5-600; (e) Ti-Eu5-600; (f) Ti-Sm5-600; (g) Ti-Eu5-700; (h) Ti-Sm5-700. Scale bars represent 50 μm.

The morphologies of the synthesized titania nanopowders were analyzed by SEM. Micrographs of the powders used in the catalysis studies are shown in Figure 4. They have the typical morphologies and variable aggregate sizes of hydrothermally synthesized titania. This is a result of hydrolysis caused by crystal water from REE nitrate precursors. To investigate the distribution of Sm and Eu in the powders, elemental mapping was performed. Sm and Eu were evenly distributed in the samples, as can be seen for the 2.5 mol % nanopowders in Figure 1. The nanosized character was confirmed by AFM imaging of Ti-Eu2.5-500 and Ti-Eu5-700 (Figures S2 and S3), with particle sizes in the range 20–30 nm for Ti-Eu2.5-500 and 30–50 nm for Ti-Eu5-700.

### Vibration Spectrometry

**Figure 5:**
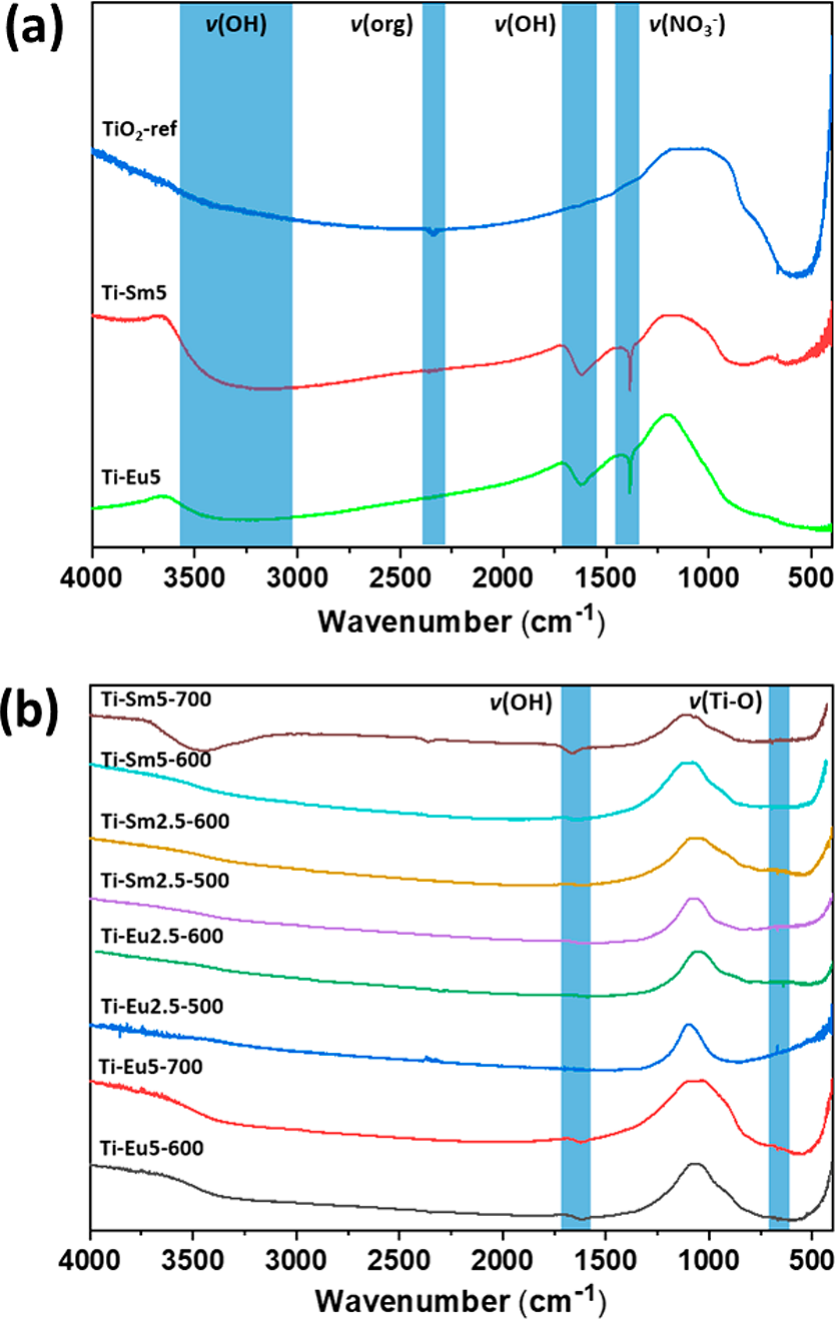
(a) FTIR spectra of amorphous Ti-Eu5 and Ti-Sm5 powders and reference titania. Interpretations of the signals are indicated. (b) FTIR spectra of the annealed titania nanopowders used for photocatalysis. Interpretations of the signals are indicated.

Annealed and amorphous titania nanopowders were analyzed by FTIR and Raman spectroscopy. The FTIR spectra for most of the annealed samples showed an absorption band around 1615 cm^–1^, which is assigned to bending of the Ti–OH bonds. The intensity of this absorption tends to decrease with increased annealing temperature, which is expected because increasing the temperature results in more condensation and dehydration of the surface OH groups. A weak absorption around 2330 cm^–1^ may be due to the formation of various organic compounds during the solvothermal reaction, and this signal appears do diminish with increasing annealing temperature (Figure 5).

The FTIR spectra of Eu- and Sm-modified titania powders before annealing contain a strong absorption line at 1384 cm^–1^, which is assigned to the presence of nitrate (Figure 5a). During annealing of the powders, the nitrate signal disappears, as is evident from Figure 5b. In the amorphous titania, the intensities of the bending OH signals at 1615 cm^–1^ and stretching for Ti–OH between 3100 and 3500 cm^–1^ are higher because of the presence of more of Ti–OH.

**Figure 6:**
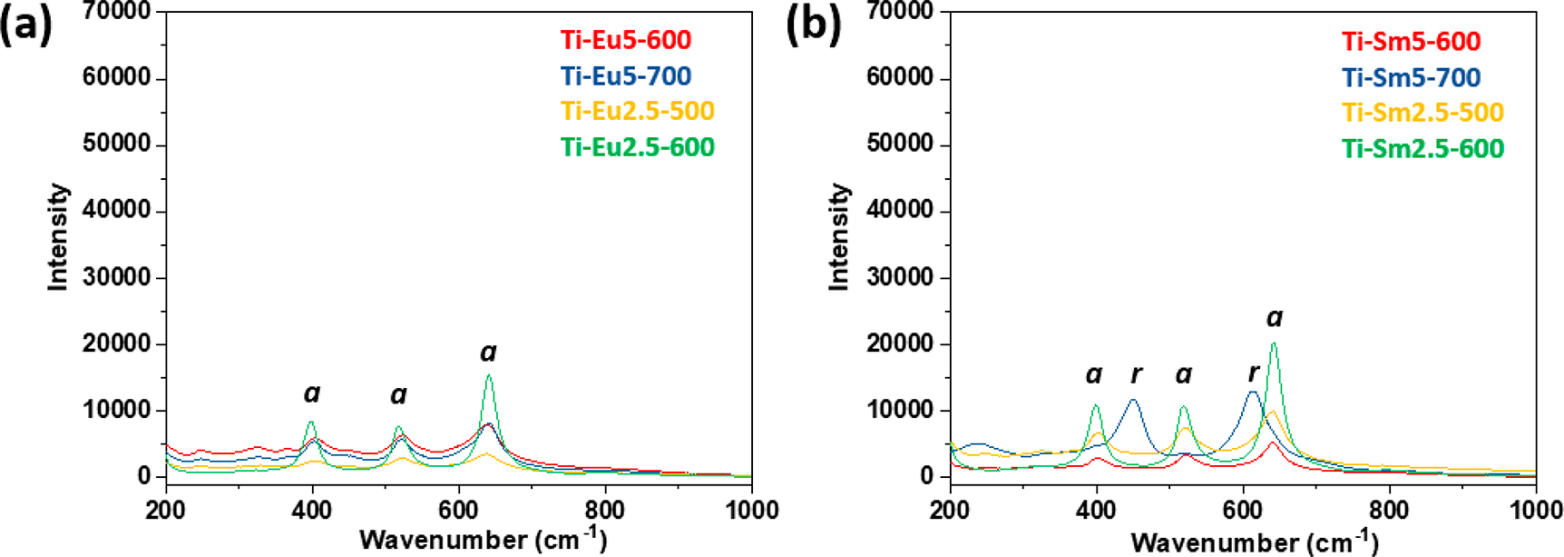
Raman spectra of titania nanopowders with the addition of (a) 2.5 and (b) 5 mol % REE. *a* = anatase and *r* = rutile.

Raman spectra were collected for the powders used in the catalytic studies (Figure 6). The spectra of the Eu-modified titania display four of the vibration modes for anatase: 199 cm^–1^ (E_g_), 401 cm^–1^ (A_1g_), 520 cm^–1^ (B_1g_), and 643 cm^–1^ (E_g_)^43^ with no traces of rutile. Three of the Sm-modified titania nanopowders display the same anatase signals, except Ti-Sm5-700, the vibration modes of which belong to the rutile phase: 449 cm^–1^ (E_g_) and 617 cm^–1^ (A_1g_).^43^ The PXRD pattern of Ti-Sm5-700 indicated anatase, with evidence of an initiated transformation into rutile. The Raman spectrum might have been collected in a small area with higher rutile content rather than being representative for the whole sample.

### PL

**Figure 7:**
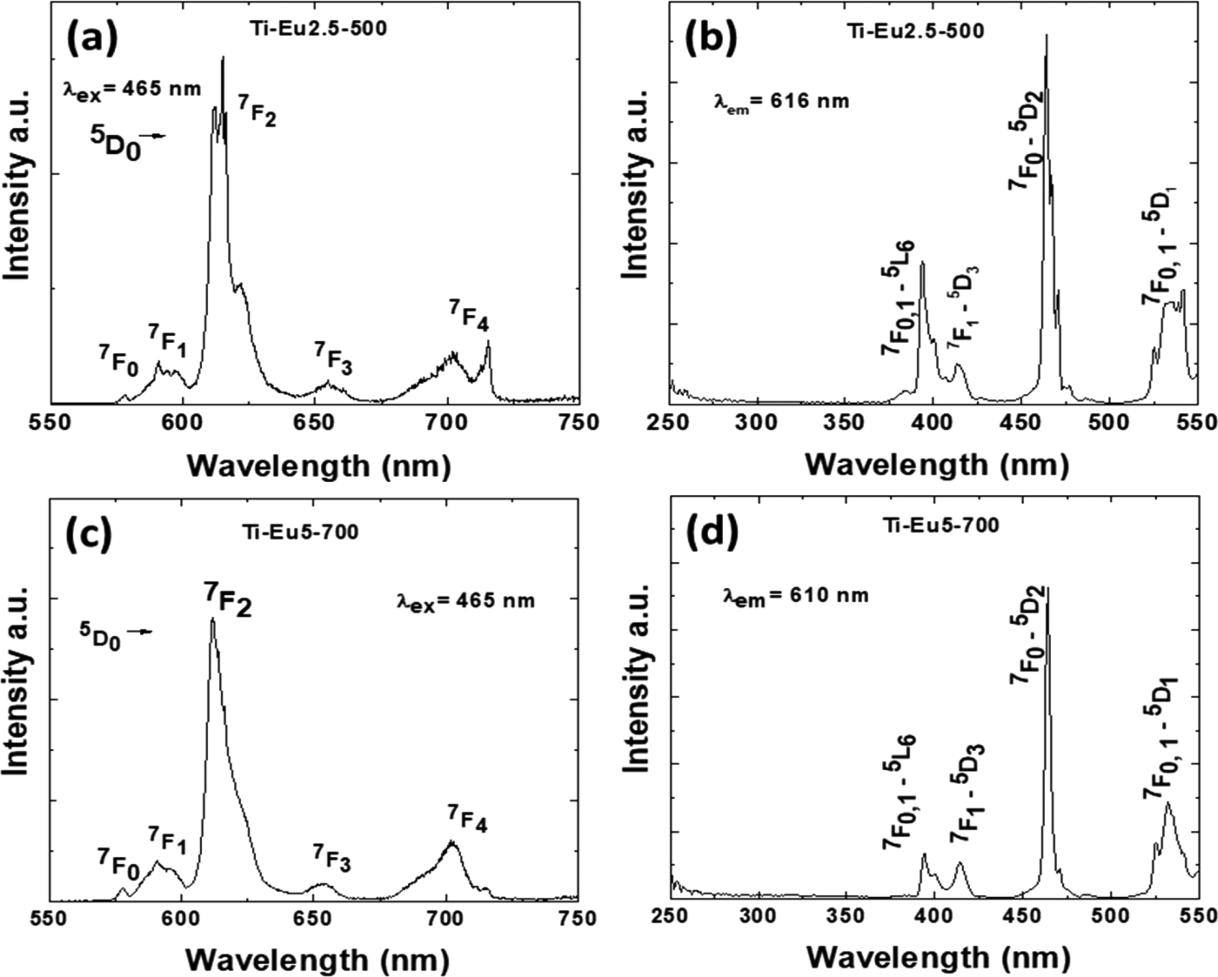
PL emission and excitation spectra of Ti-Eu2.5-500 (a and b) and Ti-Eu5-700 (c and d). The excitation and emission wavelengths are 465 and 616 nm, respectively.

**Figure 8:**
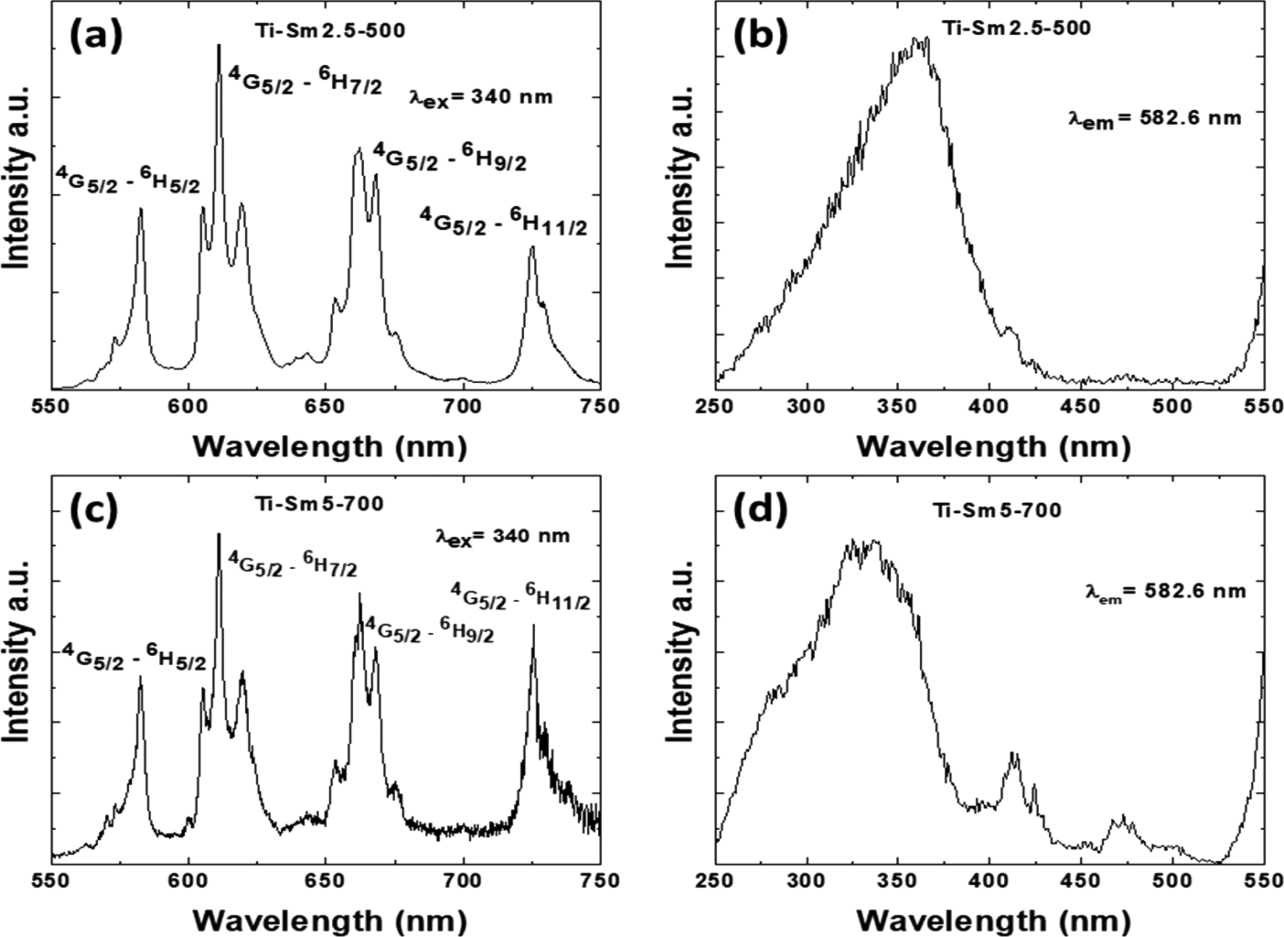
PL emission and excitation spectra of Ti-Sm2.5-500 (a and b) and Ti-Sm5-700 (c and d). The excitation and emission wavelengths are 465 and 616 nm, respectively.

Figures 7 and 8 show the room-temperature PL emission and excitation spectra of Eu- and Sm-doped TiO_2_ annealed at 500 and 700 °C, respectively. Upon excitation at 465 nm corresponding to Eu ^7^F_0_–^5^D_2_ absorption, Eu luminescence displays emission bands at 580, 590, 615, 655, and 705 nm corresponding to the Eu ^5^D_0_, ^5^D_0_–^7^F_1_, ^5^D_0_–^7^F_1_, ^5^D_0_–^7^F_3_, and ^5^D_0_–^7^F_4_ emission transitions, respectively. Some of us have recently shown by using low-temperature (80 K) site-selective excitation that Eu distributes as both discrete and continuous substitutional centers on anatase Ti^4+^ lattice sites as well as on the nanoparticle surfaces. As shown in Figure 7a, the emission of Ti-Eu2.5-500 I is a convolution of narrow and broad emissions characteristic of the substitutional center I and surface center, respectively.^44^ The Eu-TiO_2_ annealed at 700 °C, Ti-Eu5-700, presents a weaker emission with a shape similar to that of surface Eu (Figure 7b). The increase of the annealing temperature from 500 to 700 °C leads to partial migration of Eu on the surface and on the rutile lattice sites, even though the long-range cation arrangement remains of the anatase type (Figure 3).^45^ The excitation spectra monitored at 616 nm of Ti-Eu2.5-500 and Ti-Eu5-700 display only narrow Eu f–f absorptions at 394, 414, 465, 525, and 532 nm corresponding to the ^7^F_0, 1_–^5^L_6_, ^7^F_1_–^5^D_3_, ^7^F_0_–^5^D_2_, ^7^F_0_–^5^D_1_, and ^7^F_1_–^5^D_1_ absorption transitions, respectively (Figure 7b,d).

Upon excitation at 340 nm into the TiO_2_ absorption band, the Sm luminescence displays emission bands centered at 582, 610, 662, and 725 nm corresponding to the Sm ^4^G_5/2_–^6^H_5/2_, ^4^G_5/2_–^6^H_7/2_, ^4^G_5/2_–^6^H_9/2_, and ^4^G_5/2_–^6^H_11/2_ emission transitions, respectively (Figure 8a,c). Both Ti-Sm2.5-500 and Ti-Sm5-700 present similar emissions, which are close to that of the substitutional center I, as identified by Avram and co-workers.^44^ However, the emission intensity of Ti-Sm5-700 is relatively lower, which signals a partial migration of Sm on the nanoparticle surfaces and rutile Ti^4+^ lattice sites. The excitation spectra of Ti-Sm2.5-500 and Ti-Sm5-700 monitored at 582.6 nm (corresponding to the maximum of the Sm ^4^G_5/2_–^6^H_5/2_ emission transition) are dominated by the broad TiO_2_ UV absorption band, followed by weak Sm f–f absorptions in the 400–550 nm spectral range (Figure 8b,d). Different to Eu and Sm luminescences are efficiently sensitized by TiO_2_ absorption.^46^

Upon excitation above the TiO_2_ band gap, electrons are excited from the VB to CB, generating holes in the VB. The electron–hole recombination energy is nonradioactively transferred to the Eu/Sm excited states via a phonon-assisted energy-transfer process.^46^ The efficiency of sensitization by TiO_2_ absorption may depend on the relative positions of the Eu/Sm ground and excited states with respect to the VB and CB of the host lattice,^47^ which may explain the observed differences in the luminescence behavior of the Eu and Sm dopants.

### XPS

**Figure 9:**
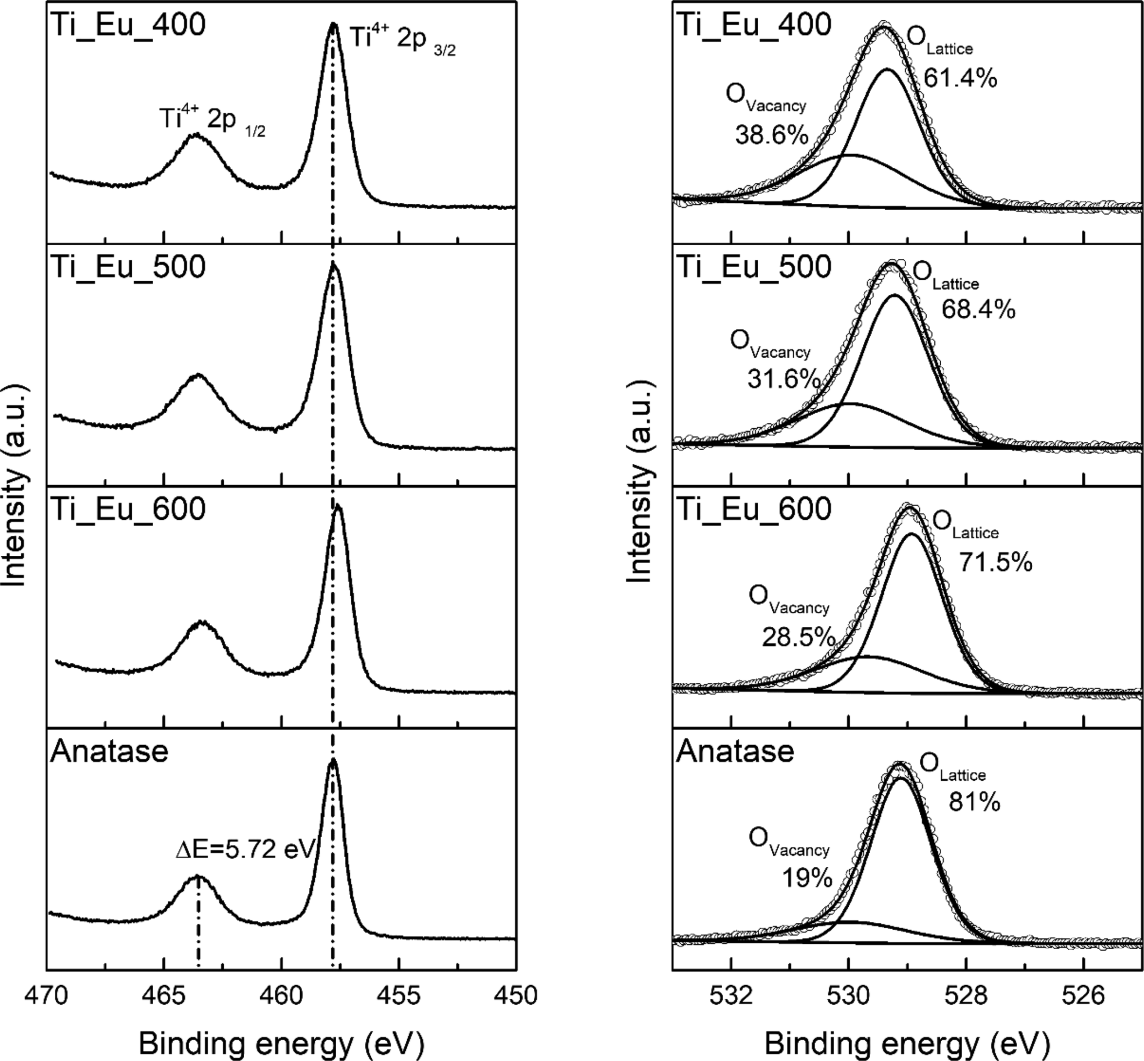
High-resolution XPS spectra of Ti 2p and O 1s of anatase and Eu-doped (5 mol %) TiO_2_ at different annealing temperatures.

The valence states of Ti 2p and O 1s for anatase and Eu-doped (5 mol %) TiO_2_ at different temperatures were evaluated by the high-resolution XPS spectra in Figure 9. Table 2 summarizes the peak positions of Ti 2p, which were obtained from the experimental data in Figure 9. Ti 2p spectra show two spin–orbit peaks with a binding energy difference of 5.72 eV, which reveals that TiO_2_ is present mainly as the Ti^4+^ oxidation state.^48^ After Eu doping, the valence state of the Ti cation can be partly reduced, as seen in the binding energy of Ti-Eu-600, shifting to a lower binding energy than that of Ti-Eu-400 (Figure 9 and Table 2). For the O 1s spectra, the peak around 529.2 eV can be assigned to oxygen from the lattice, while the O 1s spectrum peak at a higher energy (∼530 eV) is attributed to oxygen vacancies. The energy peak at ∼530 eV is stronger after Eu doping, and the O_vacancy_ area peaks of Ti-Eu-400, Ti-Eu-500, and Ti-Eu-600 increase by 19.4%, 12.6%, and 9.5%, respectively.

**Table 2:** XPS Data for Ti-Eu5 at Different Annealing Temperatures

	energy (eV)			
	anatase	Ti-Eu5-400	Ti-Eu5-500	Ti-Eu5-600
Ti 2p1/2	463.53	463.50	463.45	463.31
Ti 2p3/2	457.81	457.78	457.73	457.59

### Photocatalysis

**Figure 10:**
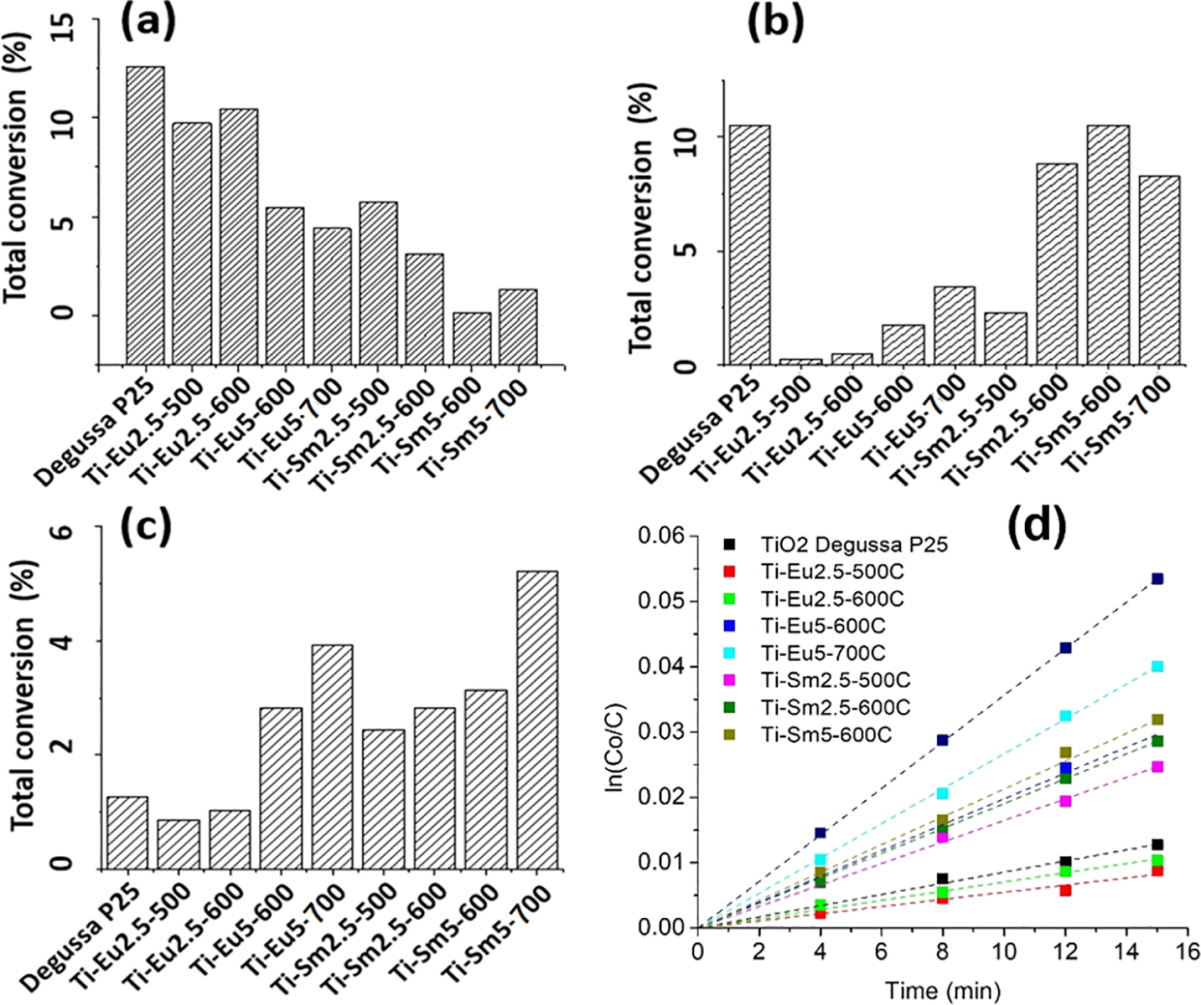
Photocatalytic degradation of TMP using titania nanopowders modified with Eu and Sm under different wavelengths: (a), 445–465 nm LEDs; (b) 510–530 nm LEDs; (c) wide-range visible lamp. (d) Pseudo-first-order reaction kinetics of TMP degradation under visible irradiation.

The photocatalytic performance of the modified powders was compared to that of the reference Degussa P25 titania (Figure 10). The degradation of 5 mM aqueous trimethylphenol (TMP) was determined spectrophotometrically after irradiation at 445–465 and 510–530 nm and a wide range of visible light for 15 min. The remaining TMP concentration was determined by high-performance liquid chromatography–UV/vis. The Sm- and Eu-modified titania showed less degradation of TMP compared to the P25 titania when irradiated with the narrow wavelengths. Interestingly, when a wide-range visible lamp was used, the modified titania had higher degradation of TMP compared to the P25 control. The overall degradation yields for all treatments were generally low but increased with prolonged radiation time. The observed differences in the catalytic activity may be explained from the PL data. The PL spectra of the Ti-Eu2.5-500, Ti-Sm2.5-500, Ti-Eu5-700, and Ti-Sm5-700 suggest the coexistence of substitutional and surface bond REEs at lower annealing temperatures for both dopants. When the annealing temperature was increased to 700 °C, the substitutional Eu^3+^ is forced to the surface while a portion of the substitutional Sm^3+^ is still present. From the similar ionic radii of the Eu^3+^ and Sm^3+^ ions, it could be expected that they would have similar substitutional behavior in the anatase lattice. However, it is indicated here that Sm^3+^ is a more effective volume dopant compared to Eu^3+^. A combination of the volume doping of anatase, broadening of the adsorption spectrum of TiO_2_ and contributing additional energy levels across the band gap, and increased defect concentration on the surface contributed to the improved activity under a wide range of visible irradiation. The different observed doping behaviors may be part of the difference in the photocatalytic activity between the different samples.

As confirmed by the plot described in Figure 10d, photocatalytic oxidation of TMP followed a pseudo-first-order reaction, ln(*C*_0_/*C*) = *kt*, where *C*_0_ is the initial concentration of the organic molecule (mmol/L), *C* its concentration at a certain reaction time, *k* the rate constant (min^–1^), and *t* the reaction time (min). The rate constants calculated on the basis of this equation are presented in Table 3.

**Table 3:** Pseudo-First-Order Rate Constants for Catalysts Treated at Different Temperatures

catalyst	rate constant, min^–1^ × 10^3^
TiO_2_ Degussa P25	0.85
Ti-Eu2.5-500	0.58
Ti-Eu2.5-600	0.69
Ti-Eu5-600	1.91
Ti-Eu5-700	2.67
Ti-Sm2.5-500	1.65
Ti-Sm2.5-600	1.91
Ti-Sm5-600	2.13
Ti-Sm5-700	3.56

According to these data, the activity is influenced by both the nature and loading of the Ln element and calcination temperature. Sm turned out to be more effective than Eu (low loading of Eu led to smaller reaction rates than the parent TiO_2_ photocatalyst), and an increase of the calcination temperature at 700 °C afforded reaction rates for higher times compared to the catalysts calcined at 500 °C. However, 700 °C is a broader, higher temperature, producing changes in the catalyst structure. These kinetic results also correlated well with the characterization results, namely, to the defect concentration induced by doping with REEs, as evidenced by the PL experiments.

The influence of the dopant and calcination temperature is more evident from the turnover frequency (TOF) values calculated by the participation of Ti and rare metals (Table 4). Accordingly, the TOF values using REE are much higher than those for Ti. However, such an efficiency can be induced only by their association with Ti. Only REEs are not effective in this photocatalytic process. The experimental error was below 1%. All of these experiments were reproduced five times.

**Table 4:** TOF Values for the Reactions Carried Out under Visible Irradiation

	TOF (min^–1^) × 10^–19^		
sample	Ti	Eu	Sm
TiO_2_ Degussa P25	3.50		
Ti-Eu2.5-500	2.55	99.6	
Ti-Eu2.5-600	3.02	117.8	
Ti-Eu5-600	8.76	166.5	
Ti-Eu5-700	12.18	231.5	
Ti-Sm2.5-500	7.15		279.0
Ti-Sm2.5-600	8.26		322.0
Ti-Sm5-600	9.49		185.2
Ti-Sm5-700	15.73		306.8

## Conclusion

The aim of the present work was to synthesize REE (Y, Eu, and Sm)-modified titania via molecular precursors. This solution–combustion approach is expected to provide an even distribution of the dopants in the final oxide phase. A bimetallic titanium–yttrium alkoxide complex was isolated as a potential molecular intermediate for Y-doped titania. This complex was very sensitive to moisture and formed anatase nuclei upon hydrolysis in water according to HRTEM. No bimetallic intermediates could be isolated for Sm or Eu. The addition of REEs was found, in accordance with previous reports, to increase the required temperature for transformation from amorphous titania to anatase and also for the anatase-to-rutile transformation. The anatase phase was stabilized against transformation to rutile until ca. 700 °C. Photocatalytic studies revealed that the REE-modified powders were more efficient photocatalysts compared to commercial P25 titania under irradiation from a wide range of visible light. PL data of the REE-modified titania indicated different substitutional behaviors for Eu^3+^ and Sm^3+^ ions, where Eu^3+^ appeared to be expelled from the anatase lattice at a lower annealing temperature than Sm^3+^. This could rationalize part of the results found in the difference in the photocatalytic activity.

## Supporting Information Available

Elemental mapping, AFM micrographs, and crystal data (PDF)

## Accession Codes

CCDC 2095265 contains the supplementary crystallographic data for this paper. These data can be obtained free of charge via www.ccdc.cam.ac.uk/data_request/cif, or by emailing data_request@ccdc.cam.ac.uk, or by contacting The Cambridge Crystallographic Data Centre, 12 Union Road, Cambridge CB2 1EZ, UK; fax: +44 1223 336033.
