# Supplementary material for: Rare-Earth-Modified Titania Nanoparticles: Molecular Insight into Synthesis and Photochemical Properties
Source: Inorg Chem. 2021 Sep 13;60(19):14820–30. doi: 10.1021/acs.inorgchem.1c02134 (PMC8493554; doi:10.1021/acs.inorgchem.1c02134)
Supplement: Supplementary file 1 — ic1c02134_si_001.pdf [file ic1c02134_si_001.pdf]

Supplementary Information

Rare-Earth Modified Titania Nanoparticles: Molecular insight into Synthesis and Photochemical Properties

Fredric G. Svensson[1], Bogdan Cojocaru[2], Zhen Qiu[3], Vasile Parvulescu[2], Tomas Edvinsson[3], Gulaim A. Seisenbaeva[1], Carmen Tiseanu[4], and Vadim G. Kessler*[1]*

[1]Department of Molecular Sciences, Swedish University of Agricultural Sciences, Box 7015, SE-75007 Uppsala, Sweden.

[2]Department of Chemistry, University of Bucharest, B-dul Regina Elisabeta, nr. 4-12, RO-030018 Bucharest, Romania.

[3]Department of Materials Science & Engineering, Box 53, Uppsala University, SE-75103 Uppsala, Sweden.

[4]National Institute for Laser, Plasma and Radiation Physics (NILPR), RO-76900 Bucharest-Magurele, Romania.

*Correspondence: Vadim G. Kessler: vadim.kessler@slu.se; Bogdan Cojocaru: bogdan.cojocaru@chimie.unibuc.ro

Elemental mapping

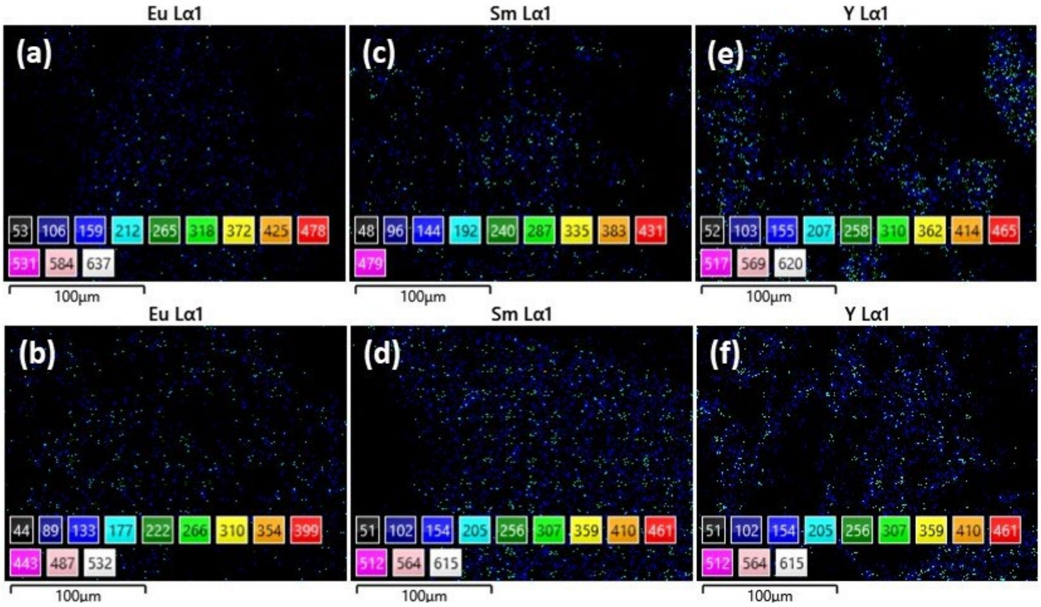

Figure S1. Elemental mapping of REE-modified titania nanopowders for Sm and Eu. (a) Ti-Eu2.5-500, (b) Ti-Eu2.5-600, (c) Ti-Sm2.5-500, (d) Ti-Sm2.5-600, (e) Ti-Y2.5-500, (f) Ti-Y2.5-600.

AFM micrographs of Eu-modified titania

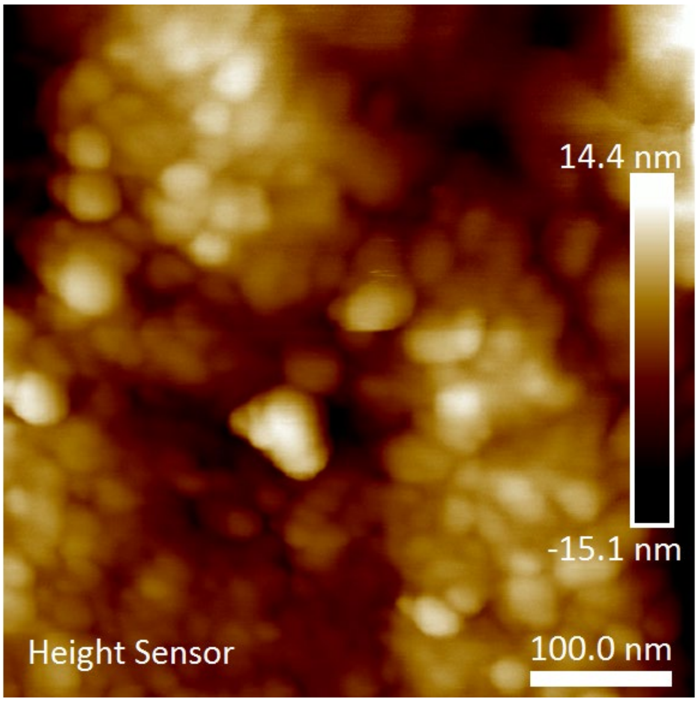

Figure S2. AFM micrograph of Ti-Eu2.5-500.

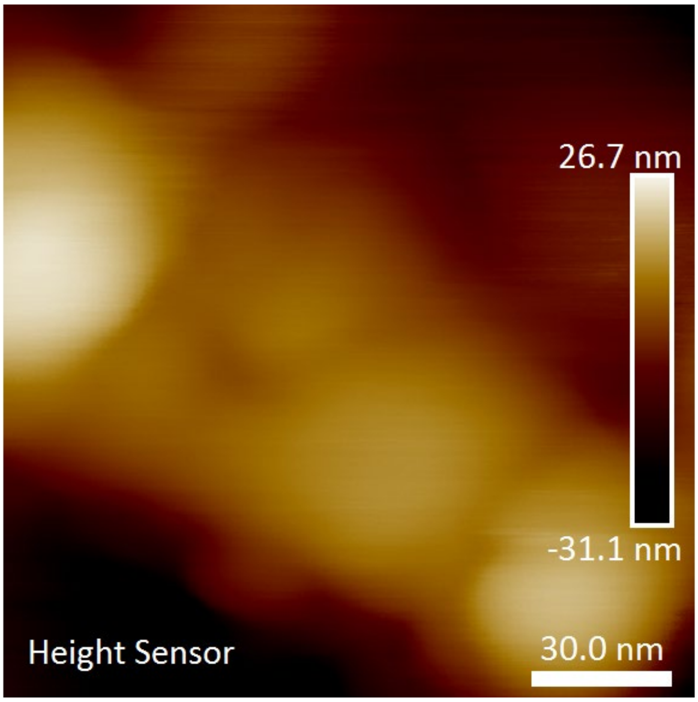

Figure S3. AFM micrograph of Ti-Eu5-700.

Supplementary Table S1. Bond lengths for Compound **1**.

**Bond**	**Length (Å)**
Y1−O3	2.308(5)
Y1−O2	2.314(5)
Y1−O1	2.322(5)
Y1−O6	2.324(5)
Y1−O400(N4)	2.333(14)
Y1−O410(N4)	2.372(13)
Y1−O41(N4)	2.379(12)
Y1−O40(N4)	2.391(13)
Y1−O152(N2)	2.315(15)
Y1−O153(N2)	2.400(15)
Y1−O151(N2)	2.412(13)
Y1−O150(N2)	2.460(12)
Ti2−O8	1.754(5)
Ti2−O11	1.759(6)
Ti2−O1	1.979(5)
Ti2−O4	2.026(5)
Ti2−O3	2.145(5)
Ti2−O2	2.200(5)
Ti1−O7	1.747(5)
Ti1−O5	1.756(6)
Ti1−O6	1.968(5)
Ti1−O4	2.024(5)
Ti1−O3	2.148(5)

Ti1−O2	2.187(5)
O1−C1	1.452(9)
O2−C3A	1.463(10)
O3−C3	1.422(10)
O4−C4	1.463(10)
C1−C1B	1.477(15)
C1−C1A	1.495(14)
O6−C6	1.451(9)
C4−C4A	1.395(14)
C4−C4B	1.486(13)
O5−C5	1.517(12)
O8−C8	1.369(12)
O11−C11	1.434(12)
O7−C7	1.420(12)
C6−C6A	1.503(12)
C6−C6B	1.542(11)
C3D−C3A	1.546(13)

Supplementary Table S1. Continued.

Bond	Length (Å)
C8B−C8	1.458(15)
C8A−C8	1.282(19)
C7−C7B	1.427(14)
C7−C7A	1.458(14)
C3A−C3B	1.445(13)
C5−C5A	1.474(13)
C5−C5B	1.510(13)
C3−C30	1.448(15)
C3−C32	1.496(14)
C11A−C11	1.468(14)
C11−C11B	1.41(2)
O15C−N2	1.192(12)
O43−N4	1.191(10)
N4−O410	1.152(15)
N4−O400	1.167(17)
N4−O40	1.347(18)
N4−O41	1.440(18)
N2−O152	1.101(18)
N2−O151	1.52(2)
N2−O150	1.43(2)
N2−O153	1.082(17)
